# Anticoagulation Management in High Bleeding-Risk ECMO in Adults

**DOI:** 10.3389/fcvm.2022.884063

**Published:** 2022-04-06

**Authors:** Stefano De Paulis, Franco Cavaliere

**Affiliations:** ^1^Department of Cardiovascular Sciences, Fondazione Policlinico Gemelli IRCCS, Rome, Italy; ^2^Università Cattolica del Sacro Cuore, Rome, Italy

**Keywords:** ECMO, cardiac surgery, heparin, bleeding, hemostasis

## Introduction

Extracorporeal membrane oxygenation (ECMO) is a life-saving therapy that has increasingly been used in recent years for the treatment of severe respiratory failure or cardiogenic shock, in the veno-venous (VV) or veno-arterial (VA) configuration, respectively (1). ECMO is a complex procedure not without significant complications, including both thrombosis and bleeding. The use of an extracorporeal circuit for cardiopulmonary support exposes blood to non-biologic, thrombogenic surfaces, and for this reason, ECMO protocols recommend systemic anticoagulation. The presence of active bleeding or a high bleeding-risk scenario is a common occurrence in the typical critically ill, ECMO-candidate patient making the choice of the anticoagulation strategy very challenging.

## ECMO Anticoagulation Guidelines

The Extracorporeal Life Support Organization (ELSO) guidelines for anticoagulation during ECMO recommend unfractionated heparin (UFH) as 50–100 units per kg bolus at the time of cannulation, followed by a continuous infusion of 20–50 units/kg/h to achieve an activated clotting time (ACT) of 180–220 s (2). Anticoagulation should ideally inhibit clot formation in the extracorporeal circuit to prevent embolism and/or ECMO dysfunction while preserving adequate procoagulant activity to avoid bleeding in the patient. The desirable hemostatic balance often proves difficult to be achieved and may become a nearly impossible task in a high bleeding-risk setting.

## Minimal or No Anticoagulation ECMO

Bleeding is a frequent complication of ECMO, with a reported incidence ranging from 27 to 60%. In adult patients with ECMO, the risk factors for bleeding have been identified: postsurgical (especially postcardiotomy) ECMO, recent trauma, type of cannulation (surgical, especially intrathoracic, and arterial at increased risk), ECMO duration, pre-ECMO coagulation abnormalities, and on-ECMO aPTT (>72 s), fibrinogen (<2 g/L), and platelets count (<38,000/mm^3^) (3, 4). The clinical spectrum of ECMO bleeding includes intracranial hemorrhage, surgical site bleeding, gastrointestinal and pulmonary hemorrhage, and cannulation site bleeding. In these scenarios, case reports and case series have reported successful management of bleeding of patients with ECMO with prolonged periods of no anticoagulation. Technological advances in ECMO circuits, oxygenators, and pumps have improved biocompatibility and theoretically reduced the risk of thrombotic complications. Consequently, reduced anticoagulation protocols have been introduced and pilot randomized trials have proven the feasibility of future randomized controlled trials of low vs. standard anticoagulation during ECMO. Based on the available data, systematic reviews, and meta-analyses offer an insight into the effectiveness of this new approach.

Olson et al. (5), in their systematic review, reported a total incidence of thrombosis of 22.9% in a group of 201 patients with ECMO without systemic anticoagulation for a median of 4.75 days and a total duration of anticoagulant-free ECMO of 304.7 days. Thrombotic events were circuit-related in 13.4% (mainly oxygenator thrombosis requiring exchange) and patient-related in 9.5% of the cases, with a predominance of arterial thrombosis in VA-ECMO. The reported incidence of circuit thrombosis during standard anticoagulation in the 2017 ELSO report is 15.6 and 22.1% in VA and VV-ECMO, respectively, and in two recent meta-analyses ranges from 12.8 to 29%. The bleeding events, during these anticoagulation-free ECMO periods, affected 32.8% of patients, the surgical site bleeding being the most common event. Of patients who bled, 27.3% were on antiplatelet and/or prophylactic dose anticoagulant. The reported incidence of bleeding during standard anticoagulation in the 2017 ELSO report is 39.4 and 51% for VV and VA-ECMO, respectively, and in two meta-analyses 29.3 and 33%.

Lv et al. (6), in their meta-analysis of low (target ACT 140–160 s) vs. standard anticoagulation ECMO, found no significant difference between the two groups in the incidence of deep vein thrombosis, pulmonary embolism, clots in the oxygenator or pump, and intracardiac thrombus. On the other hand, gastrointestinal tract and surgical site hemorrhage were significantly lower in the low anticoagulation group.

## Discussion

ECMO perturbs the balance of hemostasis inducing both a pro-thrombotic state and a bleeding diathesis. The triggering of coagulation and inflammatory cascades and platelets activation by the non-biologic circuit surface, the consequently almost universal thrombocytopenia and platelet dysfunction, and the abnormal flow-mediated loss of high-molecular-weight von Willebrand multimers and hypofibrinogenemia all contribute to clinical thrombotic and bleeding events. The standard approach to mitigate these phenomena is anticoagulation with UFH, titrated to primarily inhibit circuit thrombosis while preserving patient clotting capacity. However, ECMO frequently needs to be instituted in a high bleeding-risk context or even in a bleeding patient, making guidelines-driven anticoagulation management a real challenge.

A growing experience with low- or even no-anticoagulation protocols in bleeding settings has emerged and recent data support the adoption of reduced-intensity anticoagulation protocols. The incidence of thrombosis in the standard anticoagulation ELSO registry and other meta-analyses was comparable with the data reported by Olson et al. Data from the meta-analysis of Lv et al. confirmed the comparable thrombotic rates between standard and low anticoagulation protocols and showed a reduced incidence of bleeding events.

With the pending results of RCTs, comparing standard vs. low anticoagulation regimens for ECMO, it seems reasonable to tailor anticoagulation to the specific patient condition, allowing periods of no anticoagulation in the actively bleeding patients and shifting to low anticoagulation protocols in the case of high bleeding risk.

## Author Contributions

All authors listed have made a substantial, direct, and intellectual contribution to the work and approved it for publication.

## Conflict of Interest

The authors declare that the research was conducted in the absence of any commercial or financial relationships that could be construed as a potential conflict of interest.

## Publisher's Note

All claims expressed in this article are solely those of the authors and do not necessarily represent those of their affiliated organizations, or those of the publisher, the editors and the reviewers. Any product that may be evaluated in this article, or claim that may be made by its manufacturer, is not guaranteed or endorsed by the publisher.
